# Automated computation of arbor densities: a step toward identifying neuronal cell types

**DOI:** 10.3389/fnana.2014.00139

**Published:** 2014-11-25

**Authors:** Uygar Sümbül, Aleksandar Zlateski, Ashwin Vishwanathan, Richard H. Masland, H. Sebastian Seung

**Affiliations:** ^1^Department of Brain and Cognitive Sciences, Massachusetts Institute of TechnologyCambridge, MA, USA; ^2^Department of Ophthalmology, Harvard Medical SchoolBoston, MA, USA; ^3^Department of Electrical Engineering and Computer Science, Massachusetts Institute of TechnologyCambridge, MA, USA; ^4^Department of Neurobiology, Harvard Medical SchoolBoston, MA, USA; ^5^Princeton Neuroscience Institute and Computer Science Department, Princeton UniversityPrinceton, NJ, USA

**Keywords:** cell types, classification, retinal ganglion cells, reconstruction, stratification, laminar structures

## Abstract

The shape and position of a neuron convey information regarding its molecular and functional identity. The identification of cell types from structure, a classic method, relies on the time-consuming step of arbor tracing. However, as genetic tools and imaging methods make data-driven approaches to neuronal circuit analysis feasible, the need for automated processing increases. Here, we first establish that mouse retinal ganglion cell types can be as precise about distributing their arbor volumes across the inner plexiform layer as they are about distributing the skeletons of the arbors. Then, we describe an automated approach to computing the spatial distribution of the dendritic arbors, or arbor density, with respect to a global depth coordinate based on this observation. Our method involves three-dimensional reconstruction of neuronal arbors by a supervised machine learning algorithm, post-processing of the enhanced stacks to remove somata and isolate the neuron of interest, and registration of neurons to each other using automatically detected arbors of the starburst amacrine interneurons as fiducial markers. In principle, this method could be generalizable to other structures of the CNS, provided that they allow sparse labeling of the cells and contain a reliable axis of spatial reference.

## 1. Introduction

The classification of neuronal types is far from complete. Advances in genetic engineering for sparse and specific labeling (Gong et al., 2003; Wickersham et al., 2006, 2007; Kim et al., 2008; Chung et al., 2013; Ke et al., 2013) offer improved data acquisition and molecular identification of neuronal classes. However, the need for structural information has not diminished because what defines a true neuronal type is not clear when only molecular information is available. One challenge facing a successful classification is to ensure that every cell type is represented in the sample set. For the structural approach, dense reconstruction of tissues imaged by electron microscopy offers a solution to this completeness problem (Denk and Horstmann, 2004; Hayworth et al., 2006; Bock et al., 2011). On the other hand, electron microscopy is not yet capable of either obtaining large enough sample sets to capture the biological variability within individual cell types, or imaging cells with very large neuronal arbors. Light microscopy offers high throughput imaging and a large field of view to complement electron microscopy. However, the time-intensive tracing step represents a bottleneck of the overall program.

Recently, it was shown that neurons in the mammalian retina can achieve submicron precision in their laminar positioning (Sümbül et al., 2014). This was done by combining an arbor density formalism (Stepanyants and Chklovskii, 2005) with a neurite based registration system for sparsely labeled neurons. The ensuing arbor density classification suggests that a robust classification of all mammalian retinal ganglion cells is within reach. However, this study and many other previous attempts (Sun et al., 2002; Badea and Nathans, 2004; Kong et al., 2005; Coombs et al., 2006; Völgyi et al., 2009) depend on manual tracing of individual neuronal arbors, which is a time-intensive task. Tracing a neuronal arbor creates a “skeleton representation” of the arbor, which consists of interconnecting line segments going through the dendrites. The thickness of dendrites along the line segments is often ignored because tissue preparation artifacts can result in unreliable estimates. In contrast, a volumetric representation includes both the skeleton and the dendrite thickness along the skeleton. Here, we propose an automated method using volumetric analysis to aid the classification of neuron types. At the heart of our approach is the simple observation that while the arbor density representation in Sümbül et al. (2014) requires a precise characterization of laminar positioning, it does not utilize detailed descriptions of arbors. In particular, we demonstrate that volumetric stratification precision of neurons can match the trace-based precision in the mammalian retina. Our method is designed for sparse imaging scenarios. It does not address the problem of separating the arbors of overlapping neurons from each other, for which tracing may still be required. Kim et al. (2014) recently used volumetric analysis and semi-manual arbor reconstruction to identify bipolar and starburst amacrine cells in an electron microscopy setting.

Volumetric reconstruction of neuroanatomy from an image stack involves obtaining a digital representation of the neuronal arbor (i.e., a voxel is “white” if it belongs to the cell, and “black” otherwise), and registering this representation to other neuronal structures to achieve a comparative description. As a first step to reconstruct a sparsely labeled neuron, we use a convolutional network (LeCun et al., 1998), which is a supervised machine-learning architecture, to enhance the image quality and suppress the acquisition noise. Although robust and accurate reconstruction of neuronal morphology is still a largely unsolved problem, it has become a bottleneck only recently as a result of the advances in high-throughput imaging. The demand for automated reconstruction prompted the Digital Reconstruction of Axonal and Dendritic Morphology Challenge (DIADEM challenge) (Brown et al., 2011). The challenge helped disseminate many novel approaches (Bas and Erdogmus, 2011; Chothani et al., 2011; Narayanaswamy et al., 2011; Turetken et al., 2011; Wang et al., 2011; Zhao et al., 2011). We anticipate that some of these approaches may be preferable to the convolutional network module of our method depending on the imaging conditions. A common problem is that when labeling is not sparse enough, cells other than the neuron of interest are also reconstructed. Our solution is to apply a post-processing routine to remove extraneous objects after the initial reconstruction step.

In the mammalian retina, the dendrites of the starburst amacrine interneuron form two parallel surfaces in the inner plexiform layer, which serve as fiducial marks (Haverkamp and Wässle, 2000). When the tissue is not flattened to preserve internal structure, it assumes a wavy form under the microscope. We solve this problem by digitally flattening (unwarping) the stack with the guidance of starburst surfaces after the imaging is done. Finally, we obtain a common depth coordinate by registering the starburst surfaces from different stacks to each other.

## 2. Materials and methods

### 2.1. The dataset

We use the retinal ganglion cells (RGCs) from a recent study on the classification of retinal cell types (Sümbül et al., 2014). The dataset was obtained by confocal microscopy at a voxel size of 0.4 μm × 0.4 μm × 0.5 μm. This dataset also includes the relative positions of On and Off starburst amacrine interneurons for each RGC, by staining for choline acetyltransferase (Haverkamp and Wässle, 2000), thereby allowing a stratification analysis of RGCs based on starburst amacrine arbors. The methodological bottleneck of that study was the semi-automated tracing of RGC arbors, which required an average time of 40 min per trace with experienced tracers. The full dataset includes five strongly defined cell types, which have consistent and specific functional, molecular, and structural identifiers. We focus here on this subset, and omit the stacks where labeling is too dense (i.e., existence of many neurites in close proximity from more than one neuron) or too dim for fully automated analysis. In a few cases, the starburst surfaces were weakly stained; these were also omitted. After this culling, two neuron types did not have enough representatives for statistical analysis and were omitted altogether. The final dataset comprises 50 neurons that form three molecularly, physiologically, and structurally homogeneous cell types.

The JAM-B neurons express the junction adhesion molecule *JAM-B*, respond to offset of upward moving stimuli, and their arbors are asymmetric in the dorsal-ventral axis (in the central retina) (Kim et al., 2008). The W3 neurons express the *TYW3* gene, are sensitive to local edges, and have one of the smallest arbor sizes in the mammalian retina (Kim et al., 2010). The BDa neurons express the *FSTL4* gene, are On-Off direction sensitive, and arborize twice (Kim et al., 2010). Finally, these cell types are known to stratify at characteristic depths in the inner pexiform layer with submicron precision [distance from the On starburst surface: 15.6 μm (JAM-B), 5.5 μm (W3), 0.3 μm (BDa)—BDa neurons stratify again 0.3 μm distal to the Off starburst surface] (Sümbül et al., 2014).

### 2.2. Volumetric reconstruction of sparsely labeled neurons from manual traces

**Figure 1:**
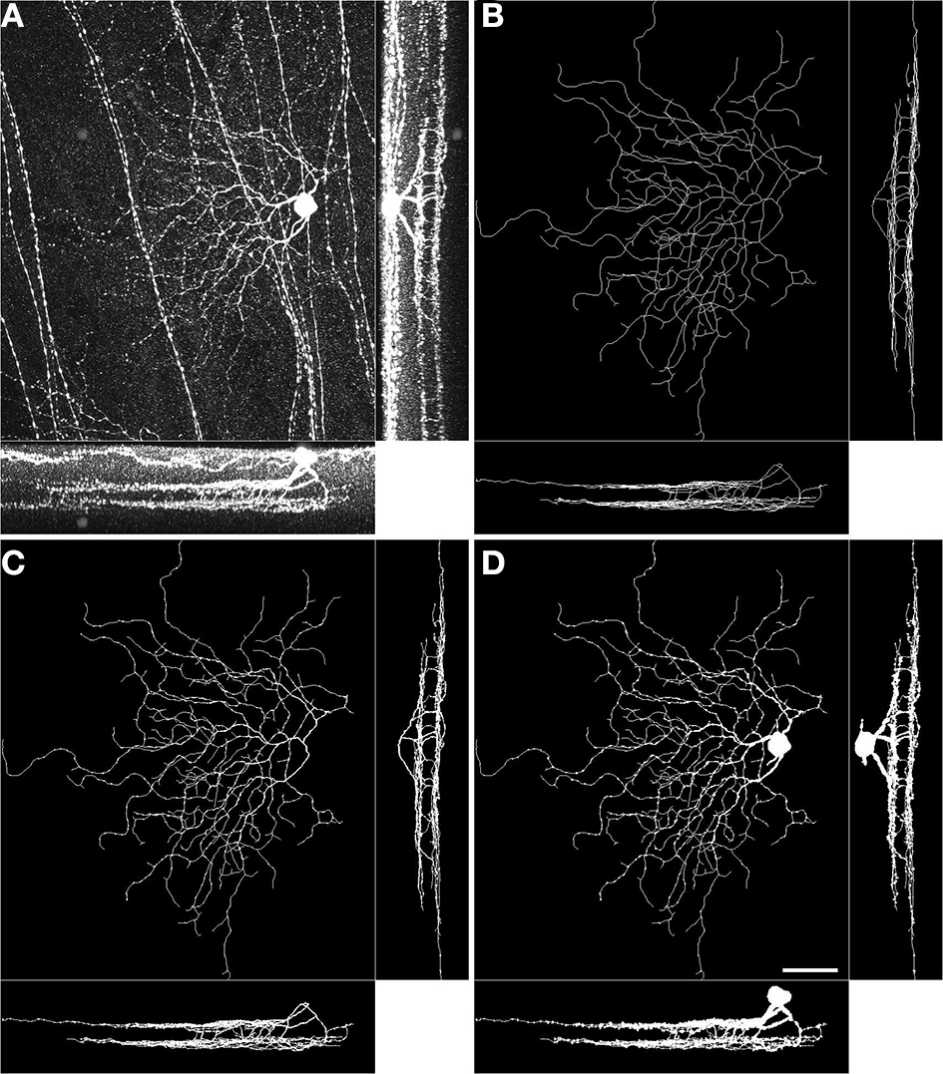
**Volumetric reconstruction of a BDa neuron starting from a trace**. Maximum intensity projections of the raw image stack **(A)**, the manually traced arbor **(B)**, the inflated trace after one round of topologically constrained inflation **(C)**, and the inflated trace after 62 rounds of topologically constrained inflation **(D)**. In each panel, large image: *xy* projection, bottom: *zy* projection, right: *xz* projection. Scale bar, 40 μm; bottom-right, *xy* projection image in panel D.

We use the concept of *simple pixel* from digital topology (Bertrand and Malandain, 1994) to probe whether neuronal mass attains the stratification precision achieved by the arbor traces (skeletons). A simple pixel is defined as a pixel that does not change the topology of the digital image when its value is flipped. (i.e., does not create/remove objects, holes, splits, mergers) Similar approaches were previously used in the reconstruction of dense electron microscopy images of neuronal tissue (Jain et al., 2010; Helmstaedter et al., 2013). Specifically, we inflate the individual traces by respecting the topology of the traces (via simple pixel characterization), and the geometry of the neurons (via thresholding the brightness values in the raw image). We use 60% of the maximum brightness value in an image stack as the threshold. We iterate the inflation process 62 times, potentially inflating by a single layer of voxels at each step so that somata as large as (62 × 2 + 1) × 0.4 μm= 50 μm in diameter are properly characterized. Algorithm 1 presents a pseudocode of the steps. The resulting three dimensional binary stacks are *seemingly* perfect characterizations of neuronal morphology based on the raw image stacks and the arbor traces (Figure 1) because they respect both the tree structure (through tracing, Figure 1B), and the dendritic widths (through inflation, Figure 1D). The caveat is that the resulting volumetric representations depend on the laborious task of (semi-) manual tracing.

**Algorithm 1:** **Pseudocode for topologically constrained inflation of a trace. Binary operations on same-size arrays are to be interpreted elementwise. ¬ (⊕) denotes negation (exclusive-or). *imdilate* dilates its first argument using its second argument as the kernel. *nnz* returns the number of nonzero (true) entries in an array. Matlab notation is used in the array on line 12**.

**Algorithm** *Inflating a trace.*
**Input:**
1. rawImage, traceImage (*m* × *n* × *p*), maximumGrowthRadius, threshold (scalar)
**Output:** volume (*m* × *n* × *p*)
2. (^*^ Initialization ^*^)
3. volume ← traceImage, target ← rawImage ≥ threshold
4. dilationKernel ←(3 × 3 × 3, binary) 6-neighborhood
5. (^*^ Growth ^*^)
6. **for** *i* ← 1 **to** maximumGrowthRadius
7. **do** mask ← imdilate(volume, dilationKernel) & ¬ volume
8. volumeCopy ← volume, previousDiff ← -1, current Dif ← 0
9. **while** currentDiff ≠ previousDiff
10. **do** differenceVoxels← mask & (volume ⊕ target)
11. **for** each (*x, y, z*) such that differenceVoxels (x, y, z) = **true**
12. **do** patch ← volumeCopy(x-1:x+1,y-1:y+1,z-1:z+1)
13. **if** simple26(patch) (^*^ Bertrand and Malandain 1994^*^)
14. **then** volume(x,y,z) ← ¬volume(x,y,z)
15. previousDiff←currentDiff
16. currentDiff← nnz(differenceVoxels)

### 2.3. Automated enhancement and post-processing of RGC arbors

Various approaches have been developed recently for automated reconstruction of neuronal morphology from sparsely labeled image stacks (Al-Kofahi et al., 2002, 2008; Schmitt et al., 2004; Zhang et al., 2007; Losavio et al., 2008; Peng et al., 2010, 2011; Srinivasan et al., 2010; Bas and Erdogmus, 2011; Turetken et al., 2011; Wang et al., 2011; Xie et al., 2011; Choromanska et al., 2012; Turetken et al., 2012; Gala et al., 2014). While these methods can capture the geometrical layout of neuronal arbors, imperfections in tissue handling and imaging (e.g., non-uniform labeling of neurites, high density labeling, low signal-to-noise ratio images) often result in topological errors such as missing branches and extraneous structures. On the other hand, blurring and projection operations are robust against local mistakes. Therefore, topological imperfections in the reconstruction may be acceptable for cell type identification purposes so long as the general morphology of a neuron is captured properly. As a first step, we use the convolutional network based enhancement of RGC arbors reported in (Sümbül et al., 2014). A convolutional network is a feed-forward network of convolutional filters whose outputs are transformed by a non-linearity (e.g., sigmoid). An advantage of such a supervised machine learning approach is that it does not have free parameters to adjust. Rather, the paradigm depends on the existence of a labeled training set through which the various parameters are automatically optimized. The network is trained to transform noisy gray-scale images of sparsely labeled neurons into cleaner binary images. Here, we improve the architecture and filter sizes, and provide an efficient implementation that does not need specialized hardware (http://www.github.com/zlateski/znn3). The resulting network has 8 layers with 8 perceptrons in each hidden layer except for the last hidden layer, which is a fully connected layer of 100 perceptrons. The filter sizes within each layer are identical and are as follows: 5 × 5 × 1, 5 × 5 × 1, 3 × 3 × 3, 5 × 5 × 1, 3 × 3 × 3, 3 × 3 × 3, 1 × 1 × 1, 1 × 1 × 1. Therefore, the overall patch size to decide whether the central voxel of the patch belongs to a neurite or not is 19 × 19 × 7 voxels (7.6 μm × 7.6 μm × 3.5 μm). The network has all-to-all connectivity between subsequent layers, and is trained by backpropagation learning LeCun et al. (1998).

When the density of labeling is not low enough, somata and neurites of other neurons appear in the image stacks. On the other hand, the reconstructed arbors may have breaks due to dim/inhomogeneous labeling. Therefore, we devise a simple post-processing routine to isolate the neuron of interest. The algorithm uses connected component analyses and basic morphological image operations (i.e., opening and dilation) to remove extraneous structures and somata. In particular, the algorithm detects the largest object in the image stack and removes the objects that are smaller than a given size and farther from the largest object than a given distance. Somata are removed by locating and removing the white regions that are large enough to fully enclose a given ellipsoid (Algorithm 2). While soma size is known to carry information on neuronal identity, it is a weak classifier (Sun et al., 2002; Coombs et al., 2006; Völgyi et al., 2009). The final image stacks may include axonal projections from other neurons, imperfectly suppressed noise, missing small branches, extraneous branches from other neurons, and splits/mergers of the neuronal arbor depending on the image quality and the sparsity of labeling in the tissue. Nevertheless, the next few subsections demonstrate that the reconstruction quality is high enough to study stratification patterns and probe neuronal identity.

**Algorithm 2:** **Pseudocode for post-processing a binary volume. Various binary operations are as defined in Algorithm 1. Matlab notation is used for brevity. *bwlabeln* returns an array the same size as its argument, where voxels are assigned different values *iff* they belong to different objects (26-connectivity). *regionVolumes* returns a list of object sizes. *bwareaopen* removes from its first argument all objects whose volumes are smaller than the second argument. *imopen* performs a morphological opening operation on its first argument using a cubic kernel whose edge length is given by the second argument**.

**Algorithm** *Post-processing*	
**Input:**	
1.	inStack (*m* × *n* × *p*), dilationRadius, sizeThreshold, searchRadius (scalar)
**Output:** outStack (*m* × *n* × *p*)	
2.	(^*^ Initialization ^*^)
3.	threshold← 0.7, conservativeThreshold← 0.5
4.	kernel← binary spherical kernel of radius dilationRadius
5.	somaKernel← binary spherical kernel of radius searchRadius
6.	(^*^ Normalize the stack ^*^)
7.	inStack← inStack-min(inStack(:)), inStack← inStack/max(inStack(:))
8.	(^*^ Binarize and dilate the stack ^*^)
9.	connectedStack← imdilate(inStack>threshold, kernel)
10.	(^*^ Retain the largest component – connected-components analysis ^*^)
11.	labels← bwlabeln(connectedStack)
12.	indices← sort(regionVolumes(labels), 'descend')
13.	inStack(labels ≠ indices(1))←**false**
14.	(^*^ Binarize the stack conservatively and remove small components ^*^)
15.	inStack← inStack>conservativeThreshold
16.	inStack← bwareaopen(inStack, sizeThreshold)
17.	(^*^ Open and dilate the stack to remove big lumps ^*^)
18.	lumps← imdilate(imopen(inStack, searchRadius), searchRadius)
19.	outStack← inStack & ¬ lumps

### 2.4. Quasi-conformal unwarping of volumetric data and laminar registration

**Figure 2:**
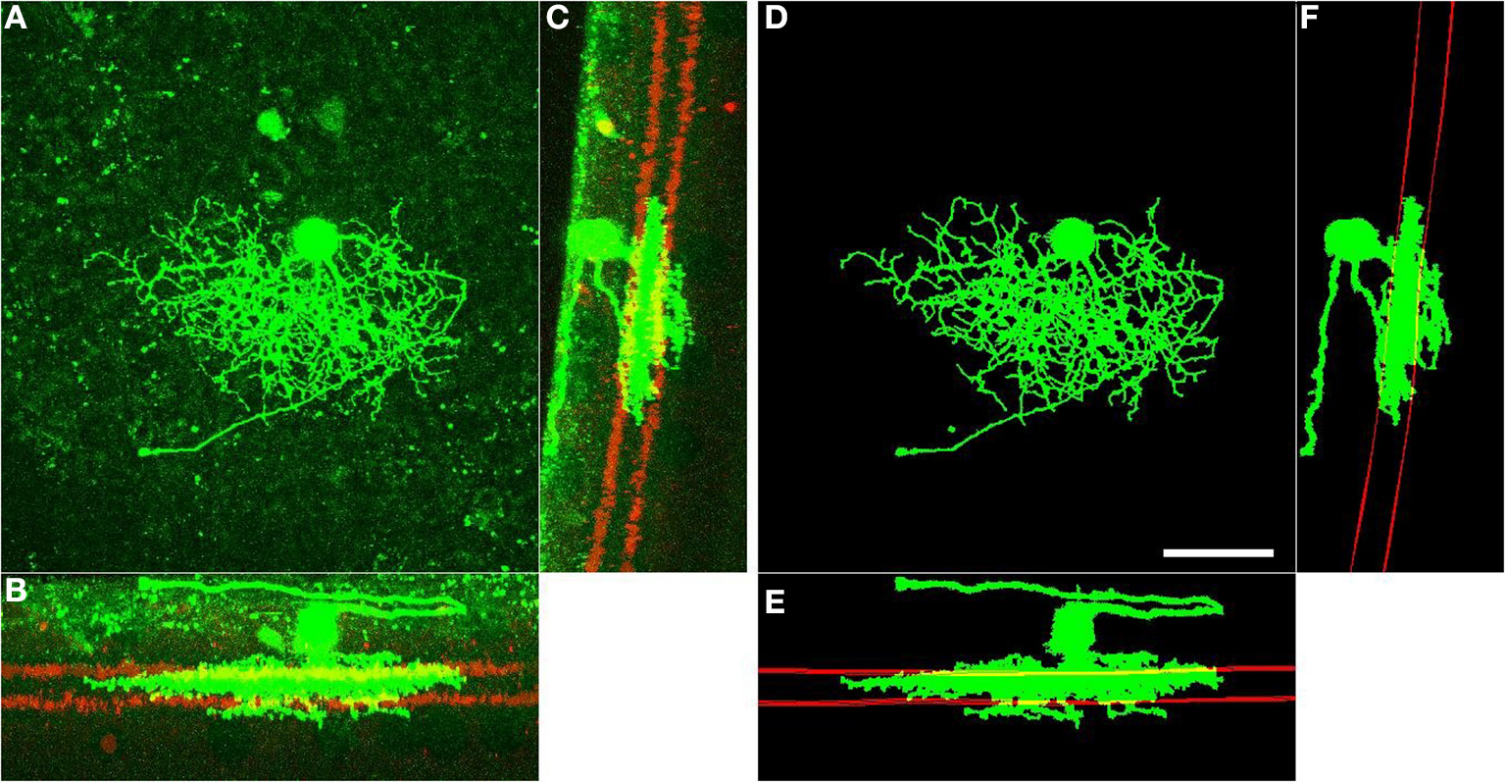
**Fully automated enhancement and post-processing of RGC arbors (green), and detection of starburst surfaces (red)**. Left: *xy* **(A)**, *xz* **(B)**, and *zy* **(C)** projections of the raw image of an RGC. Right: *xy* **(D)**, *xz* **(E)**, and *zy* **(F)** projections of the processed arbors and detected starburst surfaces of the same RGC. Starburst surfaces within a slab are shown and starburst somata are removed for better visualization. Scale bar, 40 μm; lower-right, panel **D**.

**Figure 3:**
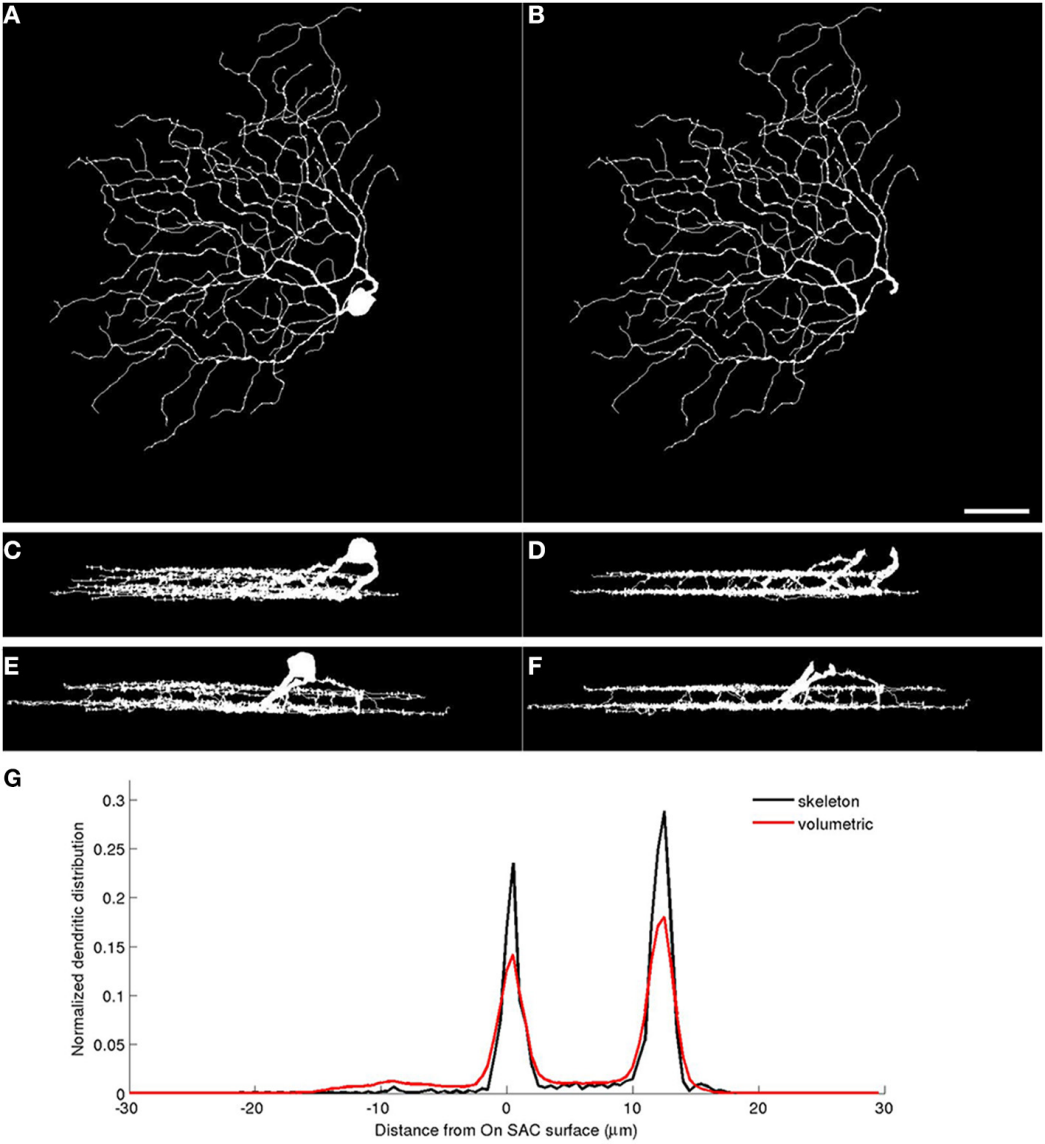
**Warping neuronal mass reveals volumetric depth profiles**. Left: *xy* **(A)**, *xz* **(C)**, and *yz* **(E)** projections of a BDa cell reconstruction obtained by inflating its trace. Right: *xy* **(B)**, *xz* **(D)**, and *yz* **(F)** projections of the reconstructions after soma removal and quasi-conformal unwarping of the white voxels. Note the depth alignment of neurites after warping. **(G)** The normalized depth profiles of the RGC based on its trace (skeleton) and trace-based volumetric reconstruction. Scale bar, 40 μm; bottom-right, panel **B**.

We use the automatically detected starburst surfaces in individual stacks as fiducial marks (Figure 2). We find quasi-conformal mappings that independently transform the detected starburst surfaces into flat surfaces as described in Sümbül et al. (2014) to maximally preserve local angles within the surfaces (Levy et al., 2002). The two flattened surfaces are registered to each other in-plane by matching the *xy* coordinates of the patch in which both starburst layers are the flattest. We extend the resulting transformation to other points in the image stack by using local polynomial approximations (quadratic in *xy*, linear in *z*). In particular we apply the transformation to individual voxels of the binary three-dimensional representation of a neuron, rather than its trace points. The transformed voxels are scaled and shifted in *z* so as to place the flattened On starburst surface at *z* = 0 μm and the flattened Off starburst surface at *z* = 12 μm. Figure 3 depicts the dramatic effect of unwarping on a BDa neuron. Finally, the histogram of depth positions of the voxels (depth profile) is obtained by gridding onto a Cartesian grid with a resolution of 0.5 μm (Figure 3D). The gridding step uses a Kaiser-Bessel kernel (Jackson et al., 1991) to maintain accuracy in laminar registration, and applies weights to individual voxels to compensate for the distance fluctuations between warped voxels. Note that if the arbor density function is obtained by blurring in *xy* only (and not in *z*), then the depth profile is the projection of the three-dimensional arbor density function.

### 2.5. Statistical measures and other metrics

The peak position of a depth profile is the signed distance from the On starburst layer at which the profile achieves its maximum value. [The On (Off) layer is located at *z* = 0 μm (*z* = 12 μm.)] For the bistratified BDa cells, a second peak position is also reported. This second peak position is defined as the depth value at least 6 μm away (half the distance between starburst layers) from the first peak position, at which the remaining profile achieves its maximum value.

We assume that the peak positions of the depth profiles of individual neurons of a given type are independent and identically distributed (i.i.d.) with *N*(μ, σ^2^). The distribution of the sample variance of *n* i.i.d. *N*(μ, σ^2^) observations is given by χ^2^_*n* − 1_(*t*(*n* − 1)/σ^2^)(*n* − 1)/σ^2^, where χ^2^_*n* − 1_(*t*) denotes the chi-squared distribution with *n* − 1 degrees of freedom. The symmetrical 95% confidence interval for σ, given the sample standard deviation *s*, is

(1) $$\left[\sqrt{\frac{(n-1)s^{2}}{X_{n-1}^{-2}(0.975)}},\sqrt{\frac{(n-1)s^{2}}{X_{n-1}^{-2}(0.025)}}\right],$$

where *X*^−2^_*n* − 1_ denotes the inverse cumulative distribution function of χ^2^_*n* − 1_.

We use the Brown-Forsythe test to infer whether the different reconstruction methods return significantly different variance values for the peak stratification position of cells of the same type.

We define the “signal” in each normalized depth profile for cells of a given type as the average normalized profile over cells of that type. Then, the “noise” in each profile is the difference of the profile from the “signal” component. We define the signal-to-noise ratio (SNR) for a cell type as the average, of the Euclidean norm of the signal divided by the Euclidean norm of the noise, over all cells of that type.

The Crest factor is defined as the peak amplitude of a profile divided by the root-mean-square value of the profile. That is, it is the ratio of the peak value to the average value. It indicates how extreme the peak is in a given depth profile. Since narrow and sharp peaks in a profile where the “background” regions are small makes it easy to detect a cell type in the presence of many cell types, we use the Crest factor as a figure of merit for the different approaches analyzed in this paper.

## 3. Results

### 3.1. Projections of volumetric data preserve the stratification precision of RGCs

**Figure 4:**
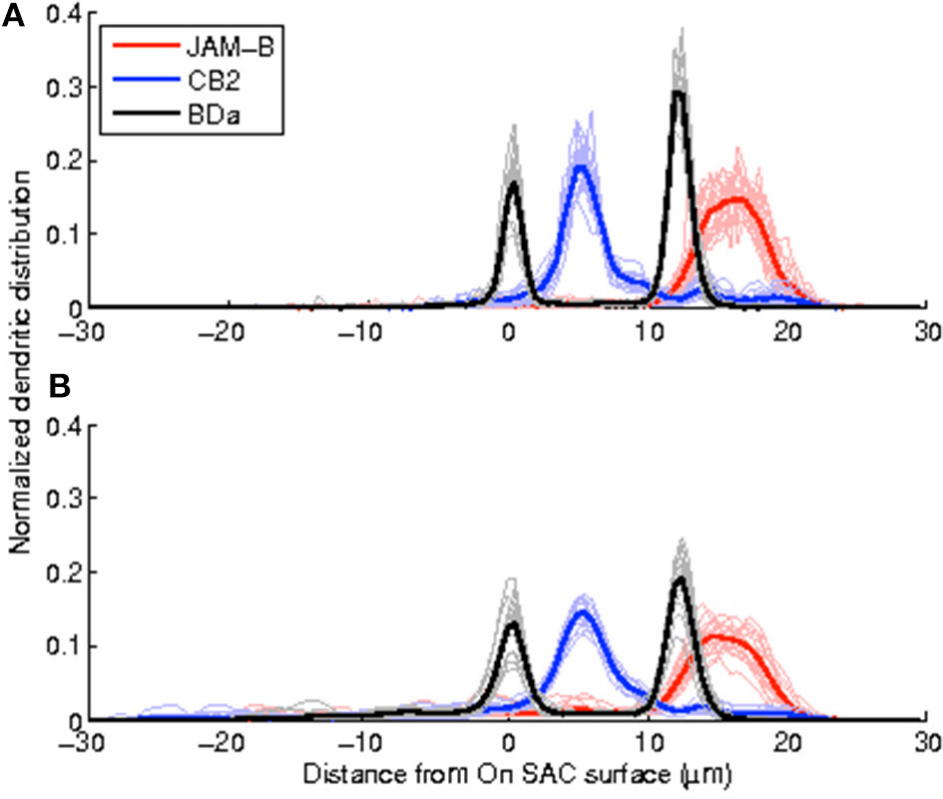
**Depth profiles of trace-based volumetric reconstructions maintain the stereotypy attained by the depth profiles of arbor traces while having lower peaks. (A)** Depth profiles of the arbor traces, **(B)**, depth profiles of the topology preserving inflations of the traces.

We obtain the volumetric reconstructions of all 50 neurons in the dataset by inflating their manually reconstructed traces as described in Algorithm 1. These volumetric reconstructions were unwarped and registered, to obtain depth profiles of all neurons in the dataset. Figure 4 shows the average profiles for each neuron type generated from the volumetric reconstructions together with the average profiles of the traces (skeletons). Two qualitative observations emerge: (i) The peak positions of the average profiles are preserved across the two methods. The distribution of mass along the skeletons of neurons preserve the peak stratification depth of the skeletons. (ii) The peaks of the normalized profile averages are lower in the volume-based approach because the branches close to the soma are typically thicker than the distal branches. Table 1 tabulates the Crest factors for both methods, and quantifies the observation that the trace profiles have slightly sharper peaks. Profiles with sharper peaks are preferable when identifying cell types in the presence of a heterogeneous dataset, similar to spectroscopy.

**Table 1:** **Mean and standard deviation values for the peak positions and norms of the depth profiles**.

	**Stratification peak**			**Crest factor**			**SNR**
	**JAMB (20)**	**CB2 (15)**	**BDa (15)**	**JAMB**	**CB2**	**BDa**	
Trace	16.13 ± 1.01	5.30 ± 0.41	0.37 ± 0.23	4.22	4.96	5.86	5.25
			12.20 ± 0.25				
Trace-based volume	15.43 ± 1.22	5.43 ± 0.26	0.40 ± 0.21	3.69	4.16	4.85	5.68
			12.37 ± 0.23				
Automated	15.70 ± 1.16	5.30 ± 0.25	0.43 ± 0.18	3.55	4.09	4.97	4.45
			12.33 ± 0.24				
Thresholded volume (^*^)	15.35 ± 1.28	5.37 ± 0.30	0.37 ± 0.23	2.79	3.86	3.82	3.24
			12.43 ± 0.18				

Values are given as mean ± SD. The number of samples (n-values) are denoted in parantheses next to the cell type names.

(*) Peaks at z < −6 μm are not considered in the calculation of stratification means and standard deviations.

The specificity of stratification peaks -not just their average- is important to be able to identify cell types. The sample standard deviations of the peak position for each cell type do not change significantly between the skeleton-based and volume-based depth profiles (Brown-Forsythe test—See Tables 1, 2 for individual *n* and *p*-values). This suggests that neurons of a given type are as precise about distributing their neuronal volume in depth as they are about distributing their skeleton-based presence.

**Table 2:** **Statistical measures of the variability in peak positions**.

**Stratification peak**			
	**JAMB (20)**	**CB2 (15)**	**BDa (15)**
**Confidence intervals**	**[0.75, 1.42]**	**[0.29, 0.61]**	**[0.16, 0.34]**
			**[0.18, 0.37]**
Trace-based vol.	0.22	0.05	0.68, 0.46
Automated	0.24	0.21	0.38, 0.72
Thresholded(^*^)	0.23	0.21	1.00, 0.11

The first row indicates the 95% confidence interval of the reported standard deviation values of the peak positions based on the traces. The remaining rows are the *p*-values of the Brown-Forsythe test of equal variance between the indicated method and the trace method. The *n*-values are denoted in parantheses next to the cell type names.

(*) Peaks at z < −6 μm are not considered.

Table 1 also tabulates the mean SNR values over the three cell types using both the trace profiles and the trace-based volumetric profiles (Methods). Higher SNR values indicate stereotypical distributions. While *n* = 3 pairs are too few to probe statistical significance with rigor, the trace-based volumetric profiles do not seem to have lower SNR values than the trace profiles. (Right-sided Wilcoxon signed rank test, *n* = 3 pairs, *p* = 0.75)

### 3.2. An automated method to probe RGC identity

**Figure 5:**
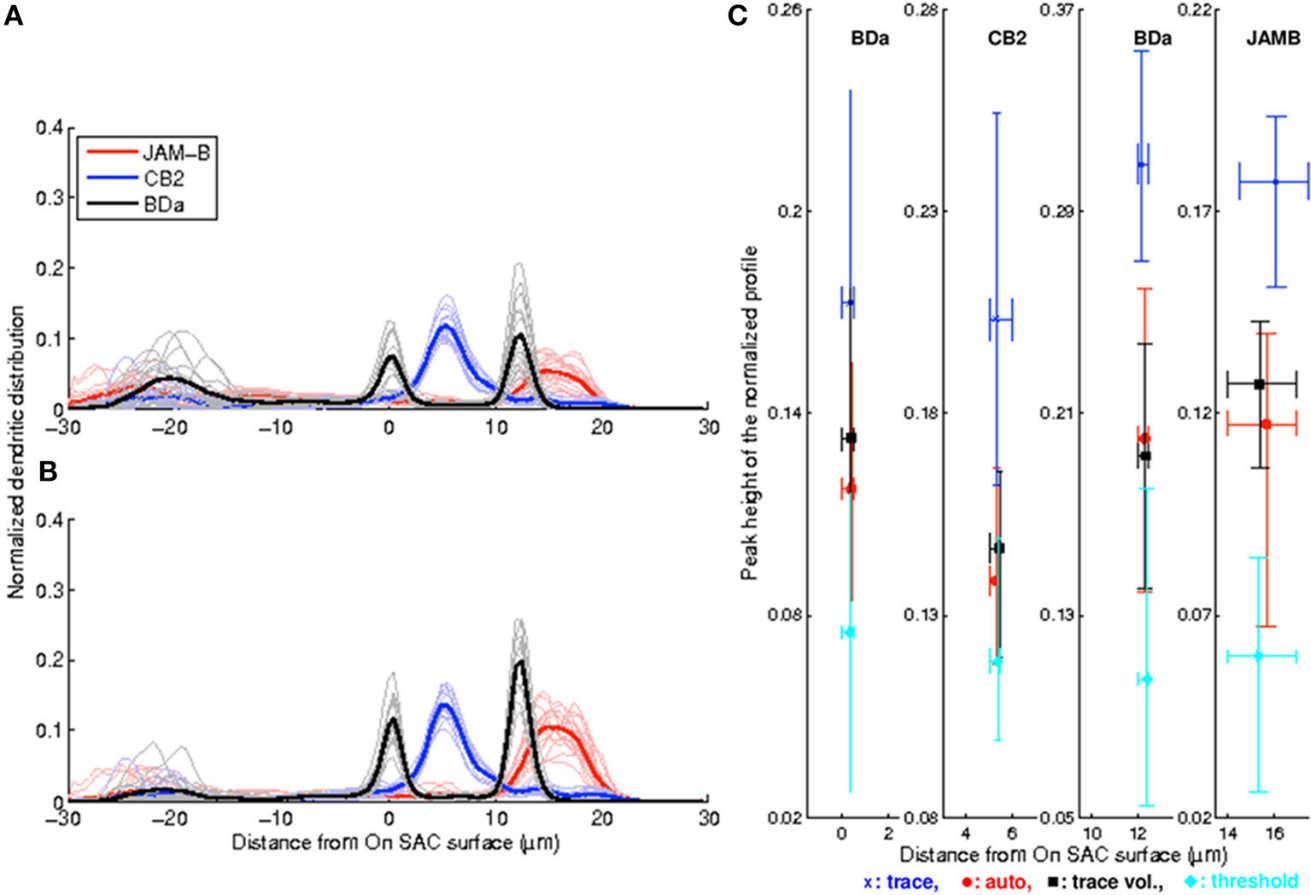
**Enhancement and post-processing enable automated peak detection and produce sharper depth profiles with higher SNR values. (A)** Normalized depth profiles obtained by thresholding the raw stack display large build-ups close to the ganglion cell layer that create spurious profile peaks and low SNR values. **(B)** Enhancement and post-processing remove objects not belonging to the neuron of interest while retaining the neuron to produce depth profiles with consistent peak positions and higher SNR values. **C**, Peak values vs. peak positions of all four methods. Symbols indicate the mean values, and the lower/upper bounds indicate the 10th and 90th percentiles. Each color indicates a different method as defined in the legend, and each panel depicts a single cell type as indicated at the top. Both peaks are shown for the bistratified BDa neurons. Peaks at *z* < −6 μm are not considered for the threshold method.

The stereotypy of the profiles of volume-based reconstructions obtained by inflating manual traces suggests that it may be possible to avoid the laborious task of manual tracing altogether for cell type identification purposes. For comparison, we begin by implementing simple thresholding: Each image stack is thresholded at 60% of its maximum brightness value. Then, somata are removed and the resulting binary stack is unwarped and registered as described in the Methods. The results are not impressive: The extraneous structures and imaging artifacts contaminate many stacks significantly. As a simple proxy, we observe that the mistakes perturb the depth profiles enough to create spurious peaks far away from the original stratification peak in 11 out of 50 stacks (Figure 5A). Therefore, this simple approach is not suitable for automation. This is especially clear for cells that stratify close to the ganglion cell layer. After removing the part of the profiles where *z* < −6 μm, we find that the stratification stereotypy of the depth profiles is essentially preserved (Tables 1, 2). Nevertheless, even in this restricted region, the presence of objects that do not belong to the neuron makes type identification harder, as reflected by the low SNR values and the Crest factors (Table 1 and Figure 5C).

While the threshold based approach may allow for cell type identification when the image stacks are sparsely labeled and have very low noise, insufficient suppression of the background noise and failure to isolate the neuron of interest from other structures prevents it from working reliably on our dataset. Therefore, we apply the convolutional network described in the Methods on the image stacks to suppress the background noise, retain the neuronal structures, and connect the occasionally disconnected neurite pieces. Subsequently, we apply the post-processing routine (Methods) to remove the extraneous structures from the image stack that are not critically close to the neuron of interest. Notably, no manual labor is used in this scheme.

A drawback is that the automated approach occasionally causes splits and mergers in the reconstruction and includes extraneous structures. On the other hand, the depth profiles—one-dimensional arbor densities that serve as proxies for the three-dimensional arbor densities—identify the stratification peaks correctly (Figure 5B). Moreover, the sample standard deviation of the peak position did not change significantly in any of the three neuron types (Brown-Forsythe test—See Tables 1, 2 for individual *n* and *p*-values). The Crest factors for this automated method are lower than those of the trace profiles, but they are roughly the same as those of the trace-based volumetric profiles. Lastly, the mean SNR value for the automated method is lower than that for the trace-based approaches, but it is higher than the threshold method's mean SNR value (Table 1).

## 4. Discussion

Identifying and providing experimental access to homogeneous cell types of nervous systems is a prerequisite to understanding the fundamental principles of brain function in health and disease. Recently, it was shown that a method using a neurite based registration system and an arbor density representation of neurons is capable of robustly identifying the mammalian RGC types in a highly heterogeneous sample set (Sümbül et al., 2014). Notably, that study relied on traces of neuronal arbors, which are time consuming to obtain. Here, we show that the spatial distribution of the arbor volume attains a stratification precision similar to that of the arbor trace. Based on this observation, we describe an automated method that can remove the time intensive tracing step in identifying cell types. We anticipate our approach to be useful in integrating structural information to studies that investigate the molecular or functional dynamics of neurons, as well as purely anatomical pursuits.

We quantify the stratification precision as the standard deviation of the peak position of the depth profiles. We do not observe significant differences between the stratification precisions of the depth profiles of the traces and the volumes obtained by inflating the traces or by our automated method (Table 2). This suggests that the depth distribution of the overall mass can be as stereotyped as that of of the skeletal mass. Another observation suggesting volumetric stereotypy is the lack of a significant difference between the mean SNR values of the normalized depth profiles of the traces and the volumes obtained by inflating the traces.

We have argued that the presented method can be useful in identifying cell types using three-dimensional arbor densities. However, we have not attempted a formal classification of the cells used in this study. While Figure 5C, Tables 1, 2 clearly suggest that such an attempt would be successful, classification becomes a hard task only in the presence of a highly heterogeneous dataset. On the other hand, considering that the automated approach can maintain the stratification precision attained by the trace based analysis and the arbor density representation in Sümbül et al. (2014) used substantial in-plane blurring (and no axial blurring), it is plausible that arbor densities generated from the output of our automated method can be classified successfully not only in the presently studied dataset of three cell types, but also in a more heterogeneous sample set.

We observe that the peak values of the normalized volumetric profiles are smaller than those of the normalized trace profiles. This can be explained by the fact that branches closer to the soma are typically thicker than the distal branches, presumably to minimize signal propagation delays while keeping arbor volume to a minimum (Chklovskii and Stepanyants, 2003).

Dim or inhomogeneous labeling of neurites, denser (not sparse enough) labeling of neurons, and high noise levels often result in imperfect reconstructions with the current state-of-the-art automated approaches. Our convolutional network implementation is not immune to such imperfections, either. Removal of failing image stacks decreases the throughput of the overall method. On the other hand, standard approaches in machine learning, such as boosting and training deeper networks with larger training data, suggest ways of increasing the throughput by providing better noise suppression and better reconstruction of arbor topology. Moreover, while other automated reconstruction methods often require manual tuning of free parameters, they can be inserted instead of our convolutional network implementation as well (Al-Kofahi et al., 2002, 2008; Schmitt et al., 2004; Zhang et al., 2007; Losavio et al., 2008; Peng et al., 2010, 2011; Srinivasan et al., 2010; Bas and Erdogmus, 2011; Turetken et al., 2011, 2012; Wang et al., 2011; Xie et al., 2011; Choromanska et al., 2012; Gala et al., 2014).

While we investigate retinal ganglion neurons in this study, our approach only assumes (i) the existence of an arbor marker specific to a cell type and (ii) a method of labeling cells sparsely in a laminar structure. Therefore, it is readily extendible to other neuron classes of the retina. In particular, the same fiducial marks (starburst amacrine cells) and very similar sparse labeling methods can be used to study the classification and co-stratification of bipolar and amacrine cell classes. The effort required to trace a neuron increases as the complexity of its arbor increases. Hence, the potential impact of our method is higher for neurons whose total dendritic lengths are larger. Cortical neurons are typically much larger than retinal neurons, and classifying them is an impending problem (Ascoli et al., 2008). Traditionally, obtaining datasets of cortical neurons that capture their diversity has been a practical challenge. However, recent advances in tissue clarification and a multiplicity of genetic or viral methods (Gong et al., 2003; Wickersham et al., 2006, 2007; Kim et al., 2008; Chung et al., 2013; Ke et al., 2013) enable high-throughput structural imaging of such neurons. Therefore, we speculate that our approach can be useful in automating the discovery and identification of cortical cell types if the two requirements mentioned above are met.

## Data sharing statement

The ZNN library is available at http://www.github.com/zlateski/znn3. The subset of trace files, the associated automatically detected starburst surfaces, the trace based reconstructions, and the software used in this study are available at http://www.github.com/uygarsumbul/volumetricRGC. The original dataset of trace files is available at http://www.github.com/uygarsumbul/rgc. Raw image stacks are available upon request.

## Author contributions

All authors contributed to the conception of the study and the editing of the manuscript. Uygar Sümbül designed and performed the analysis. Aleksandar Zlateski conceived and implemented the ZNN package. Uygar Sümbül and Ashwin Vishwanathan wrote an initial draft of the manuscript.

## Funding

We are grateful for financial support from the Harvard Neuro-Discovery Center, the U.S. Army Research Office (W911NF-12-1-0594), DARPA (HR0011-14-2-0004), NIH/NINDS, the Howard Hughes Medical Institute, the Gatsby Charitable Foundation, and the Human Frontier Science Program.

### Conflict of interest statement

The authors declare that the research was conducted in the absence of any commercial or financial relationships that could be construed as a potential conflict of interest.
